# Supplementary material for: An Efficient 2D Protocol for Differentiation of iPSCs into Mature Postmitotic Dopaminergic Neurons: Application for Modeling Parkinson’s Disease
Source: Int J Mol Sci. 2023 Apr 14;24(8):7297. doi: 10.3390/ijms24087297 (PMC10139404; doi:10.3390/ijms24087297)
Supplement: Supplementary file 1 [file ijms-24-07297-s001.zip › Figure S1.pdf]

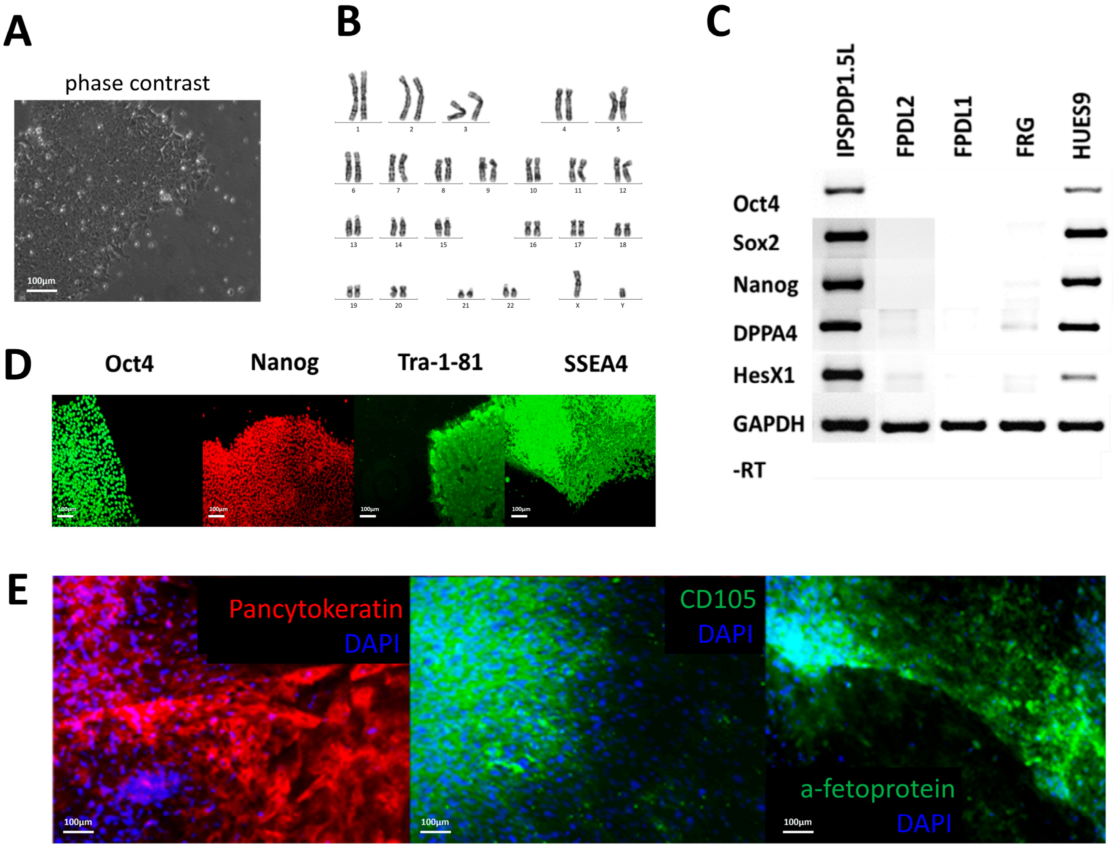

Figure S1. IPSPDP1.5L iPSC line characterization. A – cell line has morphology of typical iPSC colony, phase contrast image of representative colony, scale bar 100 µm; B - cell line demonstrated normal karyotype; C - the electrophoregram of RT-PCR products with specific primers for the OCT4, SOX2, Nanog, DPPA4, HESX1, and GAPDH genes; skin fibroblasts lines FPDL2, FPDL1 and FRG are represented as a negative control; human embryonic stem cell line HUES9 is represented as a positive control; D - cell line has positive immunofluorescent staining for key pluripotency transcriptional factor (OCT4 – green and Nanog - red), and pluripotency surface markers (TRA-1-81 and SSEA-4 - green), scale bar 100 µm; E - expression of three germ layers markers: Pancytokeratin (red), CD105 (green), and AFP (red) in embryoid bodies obtained from IPSPDP1.5L cell line, blue – DAPI, scale bar 100 µm
