# Supplementary material for: An Efficient 2D Protocol for Differentiation of iPSCs into Mature Postmitotic Dopaminergic Neurons: Application for Modeling Parkinson’s Disease
Source: Int J Mol Sci. 2023 Apr 14;24(8):7297. doi: 10.3390/ijms24087297 (PMC10139404; doi:10.3390/ijms24087297)
Supplement: Supplementary file 1 [file ijms-24-07297-s001.zip › Table S1.pdf]

Table S1. The proportion of cells in G1, S, and G2 phases of the cell cycle in mature neuron population.

	IPSRG2L (n=5)	IPSPDL2.15L (n=3)	IPSPDP1.5L (n=5)
G1	83,7 ± 3,4	88,06333 ± 7,9	91,218 ± 2,7
S	13,9 ± 3,7	9,743333 ± 6,8	7,068 ± 1,4
G2	2,242 ± 1,3	1,703333 ± 1,7	0,694 ± 0,8

Data shown as mean ± SEM.
